# Phase 1b/2a study of galunisertib, a small molecule inhibitor of transforming growth factor-beta receptor I, in combination with standard temozolomide-based radiochemotherapy in patients with newly diagnosed malignant glioma

**DOI:** 10.1007/s10637-020-00910-9

**Published:** 2020-03-05

**Authors:** Antje Wick, Annick Desjardins, Cristina Suarez, Peter Forsyth, Ivelina Gueorguieva, Tiana Burkholder, Ann Louise Cleverly, Shawn T. Estrem, Shuaicheng Wang, Michael M. Lahn, Susan C. Guba, David Capper, Jordi Rodon

**Affiliations:** 1 Clinical Cooperation Unit Neuro-oncology, German Cancer Research Center, Heidelberg University Medical Center, Im Neuenheimer Feld 400, 69120 Heidelberg, Germany; 2 The Preston Robert Tisch Brain Tumor Center at Duke, Duke University, Durham, NC USA; 3 Vall d’Hebron University Hospital and Institute of Oncology, Universitat Autònoma de Barcelona, Barcelona, Spain; 4 H. Lee Moffitt Cancer Center and Research Institute, Tampa, FL USA; 5 Eli Lilly and Company, Erl Wood, UK; 6 Eli Lilly and Company, Indianapolis, IN USA; 7 BioStat Solutions, Inc, Frederick, MD USA; 8 Department of Neuropathology, Charité – Universitätsmedizin Berlin, corporate member of Freie Universität Berlin, Humboldt-Universität zu Berlin, and Berlin Institute of Health, Berlin, Germany; 9 Department of Investigational Cancer Therapeutics, Division of Cancer Medicine, U. T. M. D. Anderson Cancer Center, Houston, TX USA

**Keywords:** Glioblastoma, Galunisertib, Radiochemotherapy, T cells, Biomarkers

## Abstract

*Purpose* Galunisertib, a TGF-β inhibitor, has demonstrated antitumor effects in preclinical and radiographic responses in some patients with malignant glioma. This Phase 1b/2a trial investigated the clinical benefit of combining galunisertib with temozolomide-based radiochemotherapy (TMZ/RTX) in patients with newly diagnosed malignant glioma (NCT01220271). *Methods* This is an open-label, 2-arm Phase 1b/2a study (*N* = 56) of galunisertib (intermittent dosing: 14 days on/14 days off per cycle of 28 days) in combination with TMZ/RTX (*n* = 40), versus a control arm (TMZ/RTX, *n* = 16). The primary objective of Phase 1b was to determine the safe and tolerable Phase 2 dose of galunisertib. The primary objective of Phase 2a was to confirm the tolerability and pharmacodynamic profile of galunisertib with TMZ/RTX, and the secondary objectives included determining the efficacy and pharmacokinetic (PK) profile of galunisertib with TMZ/RTX in patients with glioblastoma. This study also characterized the changes in the major T-cell subsets during TMZ/RTX plus galunisertib treatment. *Results* In the Phase 2a study, efficacy results for patients treated with galunisertib plus TMZ/RTX or TMZ/RTX were: median overall survival (18.2 vs 17.9 months), median progression-free survival (7.6 vs 11.5 months), and disease control rate (80% [32/40] vs 56% [9/16] patients) respectively. PK profile of galunisertib plus TMZ/RTX regimen was consistent with previously published PK data of galunisertib. The overall safety profile across treatment arms was comparable. *Conclusion* No differences in efficacy, safety or pharmacokinetic variables were observed between the two treatment arms.

**Electronic supplementary material:**

The online version of this article (10.1007/s10637-020-00910-9) contains supplementary material, which is available to authorized users.

## Introduction

Glioblastoma (GB) is the most common and aggressive brain cancer representing approximately 15% of all primary brain tumors, and about 55% of all gliomas [1]. The standard of care for patients with GB consists of maximal surgical resection followed by radiotherapy (RTX) with concomitant and maintenance temozolomide (TMZ). This therapy results in progression-free survival (PFS) at 6 months of 53.9% and median overall survival of 14.6 months [2]. Most of the therapy-responsive patients will die within a period of 2 years and the 2- and 5-year overall survival rates are 27% and 9.8%, respectively [3].

GB is characterized by persistent angiogenesis at the tumor site, and decreased peripheral immune responsiveness in patients [4]. GB microenvironment is enriched in immunosuppressive molecules such as transforming growth factor (TGF)-β that plays a specific role in cancer cell growth [5], in addition to affecting immune cell response, and endothelial cell and fibroblast differentiation [6, 7].

TGF-β is a multifunctional cytokine that is involved in a variety of cell functions including cell proliferation, migration, survival, and death that influence tumor growth in advanced forms of cancer [6, 7]. Upon binding to their ligands (TGF-β1, 2, and 3), the TGF-β kinase receptors are phosphorylated triggering phosphorylation of SMAD2 and SMAD3, and formation of SMAD complexes [8, 9].

Galunisertib is an oral small molecule inhibitor of TGF-β kinase receptor type I (TGF-β RI/ALK5) [10] and selectively inhibits the serine/threonine activity of the receptor, thereby preventing the phosphorylation of downstream proteins, SMAD2 and SMAD3 [10]. The antitumor activity of galunisertib has been demonstrated in three different in vivo tumor models; two breast cancer models, MX1 and 4 T1; and a non-small cell lung cancer model, Calu6 and fibrosis [11–13].

Based on the role of TGF-β in patients with malignant GB, evidence of antitumor effects of TGF-β inhibitors such as galunisertib (including in a monotherapy study in glioblastoma), and a favorable short- and long-term toxicity profile [14, 15], a multicenter Phase 1b/2a clinical trial was initiated to investigate the clinical benefit of combining galunisertib with standard TMZ-based radiochemotherapy (TMZ/RTX) in patients with newly diagnosed malignant glioma.

## Methods

### Patients

Eligible male and female patients were 18 years and older with histologically proven, World Health Organization Grade III (Phase 1b part only) and IV (Phase 1b/2a) malignant glioma. Patients with newly diagnosed and untreated intracranial GB including lower grade glioma, which evolved into GB were eligible. Patients with moderate or severe cardiac disease were not eligible. Adequate hematologic, hepatic, and renal function, and a performance status of ≤ 1 on the Eastern Cooperative Oncology Group (ECOG) scale were required. Concurrent use of stereotactic radiosurgery was not allowed. A biopsy or resection was required no more than 6 weeks prior to treatment and an MRI was required within 72 h after surgery; measurable or assessable disease was not required. Patients were required to begin study treatment within 2–6 weeks after surgery.

This study was conducted according to the principles of good clinical practice, applicable laws and regulations, and the Declaration of Helsinki. The protocol was approved by each institution’s review board. This study was conducted in 9 centers in 3 countries. Between April 2011 and August 2015, 101 patients entered the study but only 75 patients were enrolled and received at least one dose of galunisertib or TMZ (patients on therapy; Online Resource: Supplemental Fig. 1). All patients provided written informed consent. This trial is registered with ClinicalTrials.gov (NCT01220271).

### Study design

In Phase 1b, two dose levels of galunisertib (2 cohorts: 160 mg/day or 300 mg/day) in combination with radiochemotherapy were studied to determine the dose for the Phase 2a portion of the study (Online Resource: Supplemental Fig. 2A). In Phase 2a, eligible patients were enrolled, and randomized (3:1) to either galunisertib at 300 mg/day plus radiochemotherapy, or to a control arm of radiochemotherapy (Online Resource: Supplemental Fig. 2A). Patients received galunisertib on an intermittent dose regimen of 14 days on/14 days off for a 28-day cycle (Online Resource: Supplemental Fig. 2B).

### Study treatment

RTX consisted of 30 fractions at 1.8 to 2.0 Gy/dose (5 days a week for 6 weeks) for a total dose up to 60 Gy (Online Resource: Supplemental Fig. 2B). Galunisertib was given orally twice daily as 150 mg tablets for 14 days on/14 days off. TMZ was administered as recommended [2]. All patients received at least 6 cycles of therapy until disease progression, death, or discontinuation due to adverse events (AEs), or other reasons.

### Safety assessments

Safety was evaluated on all patients (Phase 1b and 2a) who received at least one dose of galunisertib or TMZ. Safety analyses included AE rates, laboratory and non-laboratory changes, physical examination and other safety observations including cardiac safety, such as echocardiography/Doppler, chest CT scan, and cardiac plasma markers (brain natriuretic protein, Troponin I, Cystatin C and high sensitivity C-reactive protein).

### Efficacy assessments

Primary objective of the Phase 1b study was to determine the safe and tolerable Phase 2a dose of galunisertib in patients treated concomitantly with radiochemotherapy, and the pharmacokinetics (PK) of galunisertib in combination with TMZ.

Primary objective of the Phase 2a study was to confirm the tolerability, and evaluate the pharmacodynamic (PD) effect on T-cells of galunisertib when combined with TMZ-based radiochemotherapy in patients with GB, as measured by changes in response biomarkers and their relationship to clinical benefit (overall survival [OS]). The secondary objectives were to evaluate time-to-event variables such as progression-free survival (PFS), time-to-treatment failure (TTF), time-to-tumor progression (TTP), duration-to-tumor response (DTR), overall response rate and clinical benefit rate. Galunisertib PK was also characterized (Online Resource: Supplemental Methods). Assessment of tumor response was based on Response Assessment in Neuro-Oncology (RANO) criteria [16] (Online Resource: Supplemental Methods).

### Pharmacodynamics of biomarkers

Tumor tissue and blood samples were collected at baseline and at specified times post-baseline. The baseline expression of tissue biomarkers including glial fibrillary acidic protein (GFAP), Ki67, CD3, phospho-SMAD2 (pSMAD2), and isocitrate dehydrogenase 1 (IDH1) R132H was evaluated by immunohistochemistry staining and scoring method as described previously [17] (Online Resource: Supplemental Methods).

Patients’ hematology, and expression of lactate dehydrogenase (LDH), YKL-40, and serum S100β were determined by clinical laboratory tests. Plasma TGF-β and MDC/CCL22 were measured by enzyme-linked immunosorbent assay (ELISA) (R&D systems), and multi-analyte immunoassay panel (MAIP) of 47 analytes (Myriad/RBM), respectively.

Blood samples from patients were collected and prepared for flow cytometry by Quintiles laboratories (Durham, NC) to determine the expression of CD3+ T cell subsets, such as CD4+ and CD8+, and T regulatory cells defined as CD4 + CD25 + CD127-FoxP3+. Cell staining strategy is described in Online Resource: Supplemental Methods.

## Statistical methods

Patient disposition, demographic, safety, drug-related treatment-emergent adverse events (TEAEs), and response data were summarized using patient number, frequency counts, or percentages as appropriate. The safety analysis was based on summaries of AEs reported in Common Terminology Criteria for Adverse Events (CTCAE) version 4.0, and possibly drug related.

All time-to-event variables were analyzed using the Kaplan-Meier method with 90% confidence interval (CI). Univariate Cox models were used to evaluate results for potential prognostic markers by considering their impact on OS and PFS. Continuous markers were first converted to 2-level categorical variables by dichotomizing at the median and hazard ratios between treatment arms estimated for each level.

The absolute cell number of Treg cells, CD4+, and CD8+ T cells from each patient were presented in profile plots together with the geometric mean and 90% CI at baseline, Day 42 and Day 182 for each cell type. Pair-wise t-Tests were used to estimate the change from baseline to Day 42 for T cell subsets in each arm.

## Results

### Phase 1b

A Phase 1b study was performed to determine a safe and tolerable Phase 2a dose of galunisertib in combination with radiochemotherapy in patients with GB (Online Resource: Supplemental Fig. 1). PK analysis for galunisertib was also completed at two dose levels of galunisertib (160 mg/day [*n* = 10] or 300 mg/day [*n* = 9]) given to Phase 1b eligible patients (Online Resource: Supplemental Table 2). The dose escalation of galunisertib to 300 mg/day did not increase the toxicity profile, and overlapping toxicity was not observed when combined with radiochemotherapy (Online Resource: Supplemental Table 3). The Phase 1b PK data were consistent with previous PK analyses of galunisertib (Online Resource Supplemental Fig. 4A-C). Based on overall toxicity and PK information from Phase 1b, 300 mg/day of galunisertib was selected for the Phase 2a part of the study (Online Resource: Supplemental Results).

### Patient dispositions, demographics, and baseline characteristics

**Fig. 1:**
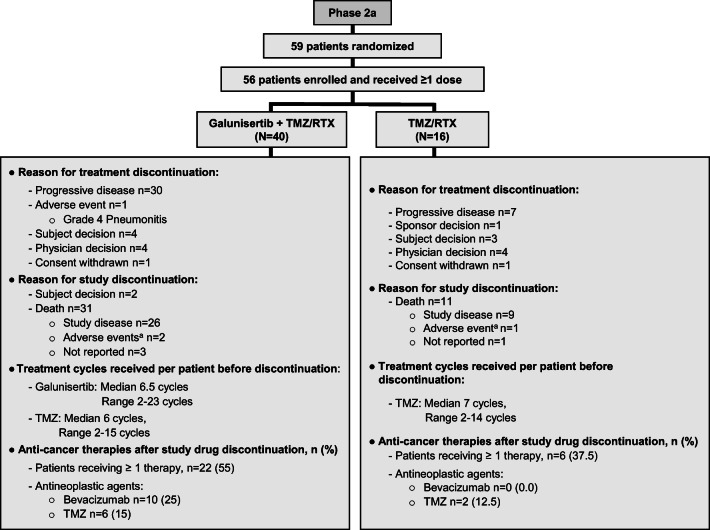
Patient dispositions from treatment. Abbreviations: TMZ = temozolomide; RTX = radiation. ^a^AEs not-related to study treatment as AEs happened >30 days after discontinuation of study treatment.

A total of 59 patients from multiple centers were randomly assigned to the Phase 2a study, and 56 patients received at least one dose of galunisertib at 300 mg/day [14] concomitantly with TMZ-based radiochemotherapy (*n* = 40) or TMZ-based radiochemotherapy (*n* = 16; Fig. 1). Among the Phase 2a patients, 38/56 (67.9%) had received complete or partial surgery as the only prior treatment for their disease; 26/40 (65%) patients who had prior surgery were treated with galunisertib plus radiochemotherapy; and 12/16 (75%) patients were treated in the radiochemotherapy alone arm (Table 1).

**Table 1:** Patient demographics and baseline characteristics

Characteristics (Phase 2a)	Gal + TMZ/RTX *N* = 40	TMZ/RTX *N* = 16	Total *N* = 56
Gender, Male	22 (55.0)	11 (68.8)	33 (58.9)
Age, years, mean, (SD)	58.7 (8.9)	57.8 (11.6)	58.4 (9.7)
Race, White	39 (97.5)	15 (93.8)	54 (96.4)
ECOG PS			
0	14 (35.0)	8 (50.0)	22 (39.3)
1	26 (65.0)	7 (43.8)	33 (58.9)
Missing	0 (0.0)	1 (6.3)	1 (1.8)
Basis of initial pathological diagnosis			
Histopathological	39 (97.5)	16 (100)	55 (98.2)
Missing	1 (2.5)	0 (0.0)	1 (1.8)
Study entry pathological diagnosis			
Glioblastoma	39 (97.5)	16 (100)	55 (98.2)
Glioma, oligodendroglioma	1 (2.5)	0 (0.0)	1 (1.8)
Prior treatment			
Surgery	26 (65.0)	12 (75.0)	38 (67.9)
Partial	14 (35.0)	6 (50.0)	20 (35.7)
Complete	12 (30.0)	6 (50.0)	18 (32.1)

To note: data given as No. (%) unless otherwise indicated

*Gal* galunisertib, *TMZ* temozolomide, *RTX* radiation, *SD* standard deviation, *ECOG PS* Eastern Cooperative Oncology Group performance status

### Treatment discontinuation

The major reason for treatment discontinuation was progressive disease (37/56 [66.1%] (Fig. 1). By the end of the study, 42/56 (75%) patients from the two treatment arms had died (galunisertib plus radiochemotherapy, 31/40 [77.5%]; radiochemotherapy, 11/16 [68.8%]). Of those who died, 35/56 [62.5%] were due to disease progression, 3/56 [5.4%] were due to AEs that started 30 days after study treatment discontinuation and cause of death was not reported for 4/56 [7.1%]). Among the 56 patients enrolled in the Phase 2a clinical trial, 28 (50%) patients moved to other anti-cancer therapies (mainly bevacizumab and TMZ) after treatment discontinuation due to disease progression (Fig. 1).

### Efficacy of treatment

**Fig. 2:**
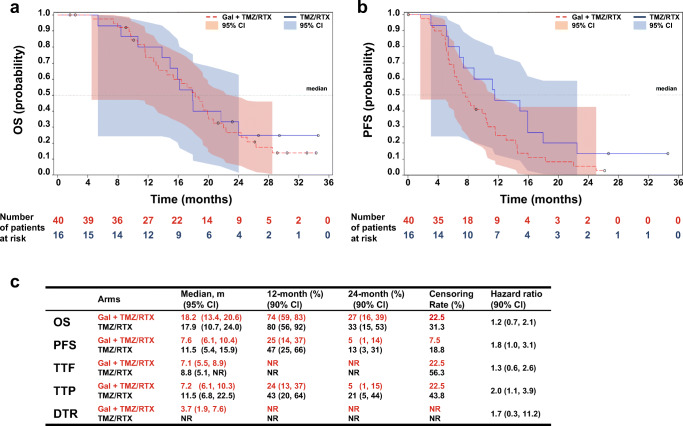
Summary of treatment responses. Kaplan-Meier estimates of OS (**a**), and PFS (**b**). Summary of OS, PFS, TTF, TTP, and DTR (**c**). Abbreviations: m = months; OS = overall survival; PFS = progression-free survival; TFF = time-to-treatment failure; TTP = time-to-tumor progression; DTR = duration of tumor response; Gal = galunisertib; TMZ = temozolomide; RTX = radiation; NR = Not reported

Overall survival was 18.2 months (95% CI: 13.4, 20.6 months) in the galunisertib plus radiochemotherapy arm compared to 17.9 months (95% CI: 10.7, 24 months) in the radiochemotherapy arm (Fig. 2a-c). Galunisertib plus radiochemotherapy, and radiochemotherapy alone had a censoring rate of 22.5% and 31.3%, respectively.

The galunisertib plus radiochemotherapy arm showed a median PFS of 7.6 months (95% CI: 6.1, 10.4 months), and the probability that PFS at 12 and 24 months, was 25% (90% CI: 14, 37%) and 5% (90% CI: 1, 14%), respectively (Fig. 2b and c). In comparison, the radiochemotherapy arm had a median PFS of 11.5 months (95% CI: 5.4, 15.9 months) (Fig. 2b and c).

Assessment of tumor response showed that 3 out of 40 patients (7.5% [90% CI: 2.1, 18.3%]) from galunisertib plus radiochemotherapy arm had a complete response compared to none in radiochemotherapy arm (Online Resource: Supplemental Table 1). Therefore, the overall disease control rate in the galunisertib plus radiochemotherapy arm was higher than in the radiochemotherapy arm (80% [90% CI: 66.8, 89.6%] vs 56.3% [90% CI: 33.3, 77.3]).

### Safety profile (AEs)

Possible all grade drug-related TEAEs (determined by CTCAE grade) occurring at a frequency of ≥ 10% of patients are described in Table 2. The most common (≥ 20%) all grade laboratory events in galunisertib plus radiochemotherapy arm were platelet count decreased (45%), lymphocyte count decreased (32.5%), and white blood cells decreased (30%). The percentage of grade 3–4 drug-related TEAEs was higher in patients treated with galunisertib plus radiochemotherapy compared to radiochemotherapy alone. Grade 3–4 most common drug-related TEAEs (≥ 10%) were decreases in platelet, lymphocyte, and white blood cell count. The most commonly reported (≥ 20%) all grade non-laboratory events in the galunisertib plus radiochemotherapy arm were fatigue (62.5%), nausea (52.5%), constipation (40%), alopecia (40%), vomiting (30%), and appetite decreased (20%).

**Table 2:** Drug-related TEAEs by CTC grade (≥10%)

	Gal + TMZ/RTX (*N* = 40)			TMZ/RTX (*N* = 16)		
	Grade 1–2	Grade ≥3	All Grade	Grade 1–2	Grade ≥3	All Grade
Laboratory Event						
Platelet count decreased	12 (30.0)	6 (15.0)	18 (45)	3 (18.8)	1 (6.3)	4 (25.1)
Lymphocyte count decreased	5 (12.5)	8 (20.0)	13 (32.5)	2 (12.5)	1 (6.3)	3 (18.8)
White blood cells decreased	8 (20.0)	4 (10.0)	12 (30)	1 (6.3)	0 (0.0)	1 (6.3)
ALT increased	4 (10.0)	3 (7.5)	7 (17.5)	2 (12.5)	0 (0.0)	2 (12.5)
Neutrophil count decreased	5 (12.5)	2 (5.0)	7 (17.5)	2 (12.5)	0 (0.0)	2 (12.5)
Anaemia	6 (15.0)	0 (0.0)	6 (15)	0 (0.0)	0 (0.0)	0 (0.0)
Non-laboratory Event						
Fatigue	24 (60.0)	1 (2.5)	25 (62.5)	6 (37.5)	0 (0.0)	6 (37.5)
Nausea	21 (52.5)	0 (0.0)	21 (52.5)	8 (50.0)	1 (6.3)	9 (56.3)
Constipation	16 (40.0)	0 (0.0)	16 (40)	4 (25.0)	0 (0.0)	4 (25.0)
Alopecia	16 (40.0)	0 (0.0)	16 (40)	3 (18.8)	0 (0.0)	3 (18.8)
Vomiting	11 (27.5)	1 (2.5)	12 (30)	5 (31.3)	0 (0.0)	5 (31.3)
Decreased Appetite	8 (20.0)	0 (0.0)	8 (20)	2 (12.5)	0 (0.0)	2 (12.5)
Headache	6 (15.0)	0 (0.0)	6 (15)	2 (12.5)	0 (0.0)	2 (12.5)
Dyspepsia	5 (12.5)	0 (0.0)	5 (12.5)	1 (6.3)	0 (0.0)	1 (6.3)
Weight decreased	5 (12.5)	0 (0.0)	5 (12.5)	1 (6.3)	0 (0.0)	1 (6.3)

To note: data given as No. (%) unless otherwise indicated

*ALT* alanine aminotransferase, *Gal* galunisertib, *TMZ* temozolomide, *RTX* radiation, *TEAE* treatment-emergent adverse events

### Correlation between biomarkers and clinical benefit

Describing and comparing the effect of galunisertib in combination with TMZ-based radiochemotherapy on major subsets of circulating immune cells, particularly Tregs (CD4 + CD25 + CD127-FoxP3+ T cells), was one of the main translational objectives of this study (Online Resource: Supplemental Fig. 3). The analysis of OS/PFS across baseline biomarkers showed no association between baseline Tregs and OS/PFS (Online Resource: Supplemental Fig. 3A and 3B). The subgroup most favoring galunisertib plus radiochemotherapy treatment compared to control was a high ratio of CD4/CD8, however it was not statistically significant.

**Fig. 3:**
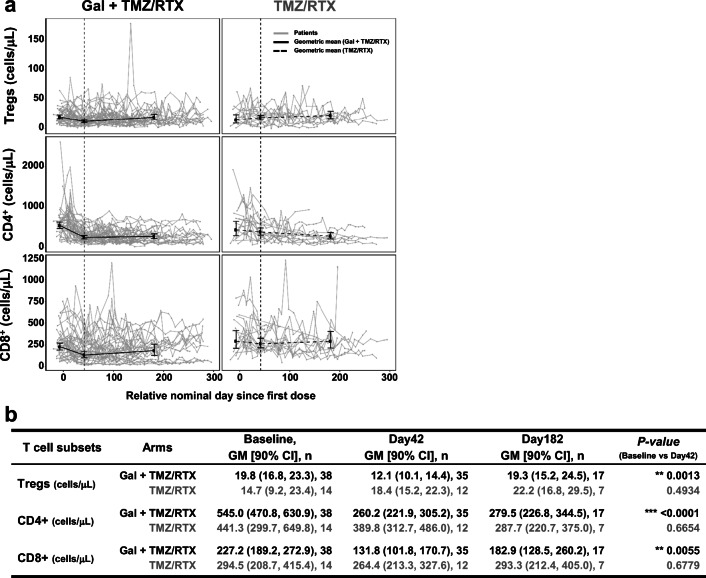
Characterization of the changes in the major T cell subsets in Phase 2a. **a** Absolute numbers of CD4 + CD25 + CD127-FoxP3+ T (Tregs), CD4+, and CD8+ T cells are reported over time from the first dose of treatment. Each gray line represents a patient. Solid and dashed lines connect the geometric mean (error bars, 90% CI) at baseline, Day 42 (end of radiation phase), and Day 182 (adjuvant phase). Vertical dotted line indicates the end of the chemoradiotherapy treatment and the beginning of the adjuvant phase. **b** Geometric mean, with 90% confidence interval, at baseline, Day 42, and Day 182. The *p* value from Pair-wise t-Test compares baseline and Day 42 within each arm. Note: Day42 = End of Radiation Phase, Cycle 2 Day 14; Day182 = Cycle 7 day 14; *P* value = Pair-wise t-Test for Comparing Baseline and Day42. Abbreviations: Gal = galunisertib; TMZ = temozolomide; RTX = radiation

Next, we evaluated the T cell counts in both treatment arms every 2 weeks for 24 weeks (Fig. 3a). Variability within and between patients was observed, but when the geometric means were assessed at baseline, Day 42 (end of radiation therapy), and Day 182 (adjuvant phase), it was apparent that CD4 and CD8 lymphocytes in galunisertib plus radiochemotherapy arm were numerically decreased during radiochemotherapy (i.e., Day 42) as previously reported for CD4 T cells [18]. A pair-wise T-test analysis comparing the geometric mean of CD4, CD8, and Treg cells at baseline and Day 42 showed a significant decrease in these cell types in the galunisertib plus radiochemotherapy arm (Baseline vs Day 42: 545.0 vs 260.2 [CD4]; 227.2 vs 131.8 [CD8]; 19.8 vs 12.1 [Treg]; Fig. 3b). After radiation, the cell counts stayed steady or slightly recovered over time when galunisertib and TMZ were given as an adjuvant treatment. After 200 days of galunisertib plus radiochemotherapy treatment, approximately 22 weeks after radiation (≈ Cycle 8) and during adjuvant treatment with TMZ, we observed two clusters of patients, one with a high number of CD8 T cells (11/16 patients), the other with a low cell number (5/16 patients) by a cutoff of around 120 cells/μl (Fig. 3a). This may suggest that galunisertib treatment may provide some protective effect on CD8 T cell population in a subset of patients.

Previous studies identified IDH1 R132H mutation as a positive prognostic marker of GB that occurs with a prevalence of almost 85% in secondary GB and approximately 5% in primary GB [19]. In the present study, the IDH1 R132H mutation was found on 6 of the 78 (8%) Phase 1b/2a-tested patients and all 6 were treated with galunisertib plus radiochemotherapy, 4 in the Phase 1b Cohort (data not shown) and 2 in the Phase 2a Cohort (Online Resource: Supplemental Table 4).

Prior to therapy, the expression of the following known tumor tissue biomarkers CD3, Ki67, GFAP, and pSMAD2 were assessed (Online Resource: Supplemental Table 4). No association between these factors and clinical outcome were identified (data not shown). In addition, the PD analysis conducted to determine the effect of galunisertib plus radiochemotherapy treatment on plasma markers (LDH, YKL-40, S100β, TGF-β and MDC/CCL22) did not find any association between the treatment and biomarkers (data not shown).

## Discussion

We here report the efficacy, safety, and PD of galunisertib combined with radiochemotherapy in newly diagnosed malignant glioma. The overall toxicity and PK results from the Phase 1b study (Online Resource: Supplemental Results) were used to determine the recommended Phase 2a dose. Additionally, PK studies done during Phase 2a showed that the plasma levels of galunisertib were not altered when combined with TMZ and radiation (Online Resource: Supplemental Fig. 4) and achieved the targeted biologically effective dose level.

While both treatments showed comparable results for median OS (18.2 vs 17.9 months), the galunisertib plus radiochemotherapy group had a shorter estimated PFS than the radiochemotherapy group (7.6 vs 11.5 months). This difference might be explained by the small number of patients in both arms or the earlier withdrawal of patients from galunisertib plus radiochemotherapy arm. For example, 55% of patients from the experimental arm were moved to subsequent therapies, versus 37.5% of patients from control received other therapies (Fig. 1).

The overall safety data across treatment arms was similar; however, the frequency of grade 3–4 toxicities was higher in the galunisertib plus radiochemotherapy arm. There was a severe case of myeloablative marrow aplasia during the first cycle of treatment in a Phase 1a patient; this finding is more likely related to the known side effect of TMZ and radiation than to galunisertib treatment [20]. Galunisertib was not associated with bone marrow side effects in preclinical toxicology studies evaluating galunisertib in human bone marrow assays or in other combination studies with chemotherapeutic agents [14].

In addition, because cardiovascular toxicities are associated with small molecule inhibitors of TGF-β signaling in preclinical toxicology studies [21], cardiac toxicity was monitored in all patients. Galunisertib treatment did not show any clinically significant cardiac safety concerns, which are consistent with previous reports for a TGF-β small molecule inhibitor [22].

Biomarker studies did not find any correlation between baseline T cell subsets (including Tregs) and OS or PFS (Online Resource: Supplemental Fig. 3A and 3B). As reported by others, the CD4+, CD8+, and Treg cells count at 10 weeks post radiochemotherapy treatment were numerically decreased [23, 24]. The longitudinal analysis shows early decrease of CD4+ and CD8+ T cell counts during radiation, followed by a steady phase or a slight recovery in these cells over time within the galunisertib plus radiochemotherapy arm, while the pattern of CD4+, CD8+, and Treg cell counts were steady over time in the radiochemotherapy arm during both radiation and post-radiation phases. In other diseases, such as lung cancer, transient decreases in CD8+ T cells followed by an increase is associated with better OS [25]. Hence, the relevance of our observation needs further examination in order to decide whether such a response would be also expected in GB patients.

An exploratory analysis was performed to examine whether any two clusters of patients with respect to OS and PFS emerged after 200 days of galunisertib plus radiochemotherapy treatment (Fig. 3a). This limited analysis set showed that the 5 patients with a low number of CD8+ T cells had a mean OS of 25.5 months and mean PFS of 14.4 months, and the 11 patients with a higher number of CD8+ cells had a mean OS of 19.4 and mean PFS of 13.1 months. However, these observations need further confirmation in larger cohorts of patients.

Furthermore, we found no association between OS and MDC/CCL22 contrary to reports for second line treatment of GB patients [26]. It is possible that baseline levels of MDC/CCL22 are different between first and second line patients. Additionally, we found no association between pSMAD2 levels in tumor tissue and OS. The presence of CD3+ T cells in tumor tissue was not associated with OS changes. These findings are different from those reported for the second line patients treated with galunisertib [17].

TGF-β is a major driver of glioma progression, via its role in tumor cell proliferation and invasion, angiogenesis, and immune suppression within the tumor microenvironment [27]. Blocking TGF-β signaling by inhibition of its receptor TGF-β RI is one strategy for abrogating its pro-tumorigenic effects. Galunisertib is one of the only drugs in development designed to specifically target TGF-β RI; it is furthest along the clinical trial pipeline [28], not only in the setting of recurrent glioma/GB (in combination with TMZ/RTX in the current study and in combination with lomustine in [15, 26]), but also for other solid tumors. Galunisertib is currently being evaluated in Phase 1/2 or Phase 2 studies in combination with immune checkpoint inhibitors [29, 30], sorafenib [31], and gemcitabine [32]. In a Phase 2 study in patients with pancreatic cancer, the first line treatment of galunisertib in combination with gemcitabine resulted in an OS benefit of 8.9 months compared to 7.1 months for patients receiving gemcitabine alone (hazard ratio [HR], 0.79; 95% CI, 0.59–1.09) [32]. In HCC patients with elevated alpha-fetoprotein prior to treatment and who had previously progressed on sorafenib or were considered not eligible to receive sorafenib, galunisertib monotherapy achieved a median OS of 7.3 months (95% CI, 4.9–10.5). OS was longer for those patients who showed reduced alpha-fetoprotein (>20% from baseline) compared to non-responders (21.5 months vs 6.8 months) [33]. While these signals were encouraging, the sponsor discontinued future clinical development for galunisertib in mid-2017 [34]. Other TGF-β RI inhibitory drugs in early clinical development (Phase 1) include LY3200882 [35] and vactosertib [36], however there are no published reports of efficacy of these compounds as of yet. In contrast to these small molecule approaches, large molecule development has had advances, including M7824 (bintrafusp alfa), which is currently being evaluated in registration studies, including for NCSLC [37].

In conclusion, the combination of galunisertib with standard radiochemotherapy did not accentuate the toxicity profile of the radiochemotherapy. Even though survival probability was unchanged between the two treatments and PFS was reduced in the galunisertib plus radiochemotherapy arm, the disease control rate was higher in the galunisertib plus radiochemotherapy treatment when compared to radiochemotherapy treatment alone. Due to new R&D priorities, Eli Lilly discontinued the development of galunisertib in 2017 [34].

## Electronic supplementary material

ESM 1 (PDF 661 kb)
