# Supplementary material for: Phase 1b/2a study of galunisertib, a small molecule inhibitor of transforming growth factor-beta receptor I, in combination with standard temozolomide-based radiochemotherapy in patients with newly diagnosed malignant glioma
Source: Invest New Drugs. 2020 Mar 5;38(5):1570–9. doi: 10.1007/s10637-020-00910-9 (PMC7497674; doi:10.1007/s10637-020-00910-9)

Electronic Supplementary Material–Online Only

Phase 1b/2a study of galunisertib in combination with standard temozolomide-based radiochemotherapy in patients with newly diagnosed malignant glioma

Antje Wick*, Annick Desjardins, Cristina Suarez, Peter Forsyth, Ivelina Gueorguieva, Tiana Burkholder, Ann Louise Cleverly, Shawn T. Estrem, Shuaicheng Wang, Michael M Lahn, Susan C Guba, David Capper, and Jordi Rodon

*Corresponding Author:

Dr. Antje Wick, MD

Heidelberg University Medical Center, Clinical Cooperation Unit Neuro-oncology,

German Cancer Research Center

antje.wick@med.uni-heidelberg.de

+49 (0) 6221-56/7588

Supplemental Methods:

Immunohistochemistry

Tumor specimens from patients' original diagnostic tumor sample were formalin fixed, and then paraffin embedded to prepare 5-μm sections for staining. The most recent tumor specimens were used for the central pathology review. Tissue staining and examinations were performed in a blinded fashion. The pathological results were entered into a database for subsequent comparison to the clinical data. Tissue histological analysis was performed as previously described [1]. Briefly, Hematoxylin and Eosin (H&E) staining was performed to assess the general anatomical phenotype of tumor samples. The baseline expression of tissue biomarkers such as glial fibrillary acidic protein (GFAP), Ki67, CD3, phospho-SMAD2 (pSMAD2), and isocitrate dehydrogenase 1 (IDH1) R132H was evaluated by IHC staining and scoring method developed at the neuropathology laboratory at the University Clinic of Heidelberg, Germany.

Flow cytometry

Briefly, after red blood cell lysis, cell surface was stained with the following antibodies from BD biosciences: CD3-V450 (clone UCHT1, mouse IgG1, κ), CD4-V500 (clone RPA-T4, mouse IgG1, κ), CD8-PerCP-5.5 (clone SK1, mouse BALB/c IgG1, κ), CD127-PE (clone HIL-7R-M21, mouse IgG1, κ), and CD25-APC (clone 2A3, mouse BALB/c IgG1, κ). FoxP3 expression was determined by intracellular staining using the FoxP3 Fix/Perm buffer kit, and FoxP3-AlexaFluor 488 (clone 259D/C7, mouse IgG1) from BD biosciences.

Assessment of tumor response

Assessment of tumor response was based on Response Assessment in Neuro-Oncology (RANO) criteria [2]. The overall response and clinical benefit rates based on RANO criteria were estimated for each treatment arm by dividing the total number of confirmed responders (CR and PR), or patients experiencing benefit by the number of patients who received at least 1 dose of study treatment. A patient with a confirmed response, partial response (PR), or stable disease (SD) was considered to have received a benefit from the treatment.

Supplemental Results

Dose reductions

During the first 3 Cycles of treatment in Phase 2a, 6 patients from the galunisertib plus radiochemotherapy cohort had dose reductions in galunisertib due to AEs such as wound infection (2 patients; Grade 3), vomiting (Grade 1), constipation (Grade 2), hydrocephalus (Grade 3), seizures (Grade 3), and alanine aminotransferase increased (Grade 3) (data not shown). The most common AE causing a dose reduction in TMZ was a decrease in platelet count in both the galunisertib plus radiochemotherapy arm and the radiochemotherapy alone arm (data not shown).

Assessment of tumor response

Among the 40 patients treated with galunisertib plus radiochemotherapy, 3 patients had a best overall response (BOR) of complete response (CR), and none had partial response (PR) representing an overall response rate of 7.5% (90% CI: 2.1, 18.3%) (Supplemental Table 1). Of the 40 patients, 29 (72.5%; 90% CI: 58.6, 83.7%) had a BOR of stable disease (SD). In the radiochemotherapy arm, none of the 16 patients reached a complete or partial response and 9 patients (56.3%; 90% CI: 33.33, 77.3%) had a BOR of SD. Therefore, the overall clinical benefit (CR+PR+SD) rate in the galunisertib plus radiochemotherapy arm was higher than in the radiochemotherapy arm (80% [90% CI: 66.8, 89.6%] vs 56.3% [90% CI: 33.3, 77.3]). Of the remaining patients, a similar percentage of patients had a BOR of PD in each arm: 7 (17.5%; 90% CI: 8.5, 30.4%) and 3 (18.8%; 90% CI: 5.3, 41.7%) in the galunisertib plus radiochemotherapy and radiochemotherapy arms, respectively. Four (25%) had an unknown status in the radiochemotherapy arm.

Pharmacokinetics (PK) for Phase 1b and 2a

The PK parameters of galunisertib from Phase 1b Cycles 1, 2, and 3 were determined by a standard noncompartmental methods of analysis using WinNonlin (supplemental Fig. 4a-c). The

PK analysis was conducted on each patient who had completed at least 2 days of sampling. Individual plasma concentration versus time profiles and individual and summary statistics of PK parameters for Phase 1b patients were carried out (Supplemental Fig. 4). To determine a potential difference in galunisertib effect in combination with radiochemotherapy (Cycle 1 and 2) or alone (Cycle 3), a PK non-compartmental analysis was performed for 18 patients (10 patients treated with 160 mg/day and 8 patients with 300 mg/day) in Phase 1b (Supplemental Fig. 4a). The maximum observed drug concentration (C_{max}), the time of maximum observed drug concentration (t_{max}), and the area under the plasma concentration versus time curve from time 0 extrapolated to infinity [AUC (0-∞)] data showed that galunisertib exposure was not altered when combined with TMZ (Supplemental Fig. 4a). The median value of the individual dose normalized AUC (0-∞),ss across the first 3 treatment cycles were similar (Supplemental Fig. 4b).

The estimation of population PK parameters of galunisertib in Phase 2a was carried out by using nonlinear mixed effect modeling (NONMEM) approach to analyze the plasma concentration of galunisertib versus time after dose normalization together with demographic factors such as weight and age. (Supplemental Fig. 4c). In Phase 2a, there were 465 observations from 40 patients across Cycles 1 and 2. There were also 42 observations without recorded dosing that were excluded and 74 below limit of quantification samples. Phase 1b and Phase 2a PK data for all days and cycles as defined in the PK sampling schedule were combined in a dataset. The observations were dose normalized and plotted by cycle in Supplemental Fig. 4c. The PK results showed no obvious difference when galunisertib was administered in combination with radiochemotherapy with TMZ (Cycle 1 and 2) and with galunisertib monotherapy (Cycle 3).

Report of Phase 1b study

Out of the 101 patients who entered in the clinical trial, 19 patients were enrolled in Phase 1b part of this trial, and then distributed in two cohorts to receive at least one dose of galunisertib at 160mg/day (n=10) or 300mg/day (n=9) combined with standard radiochemotherapy (Supplemental Fig. 1). Eligible Phase 1b patients (see methods) included those with World Health Organization Grade III malignant glioma (such as anaplastic astrocytoma, anaplastic oligoastrocytoma, and anaplastic oligodendroglioma along with WHO Grade IV patients (Supplemental Table 2).

All 19 patients (12 male patients [63.2%], and 7 female patients [36.8%]) were white with a mean age of 54.8 years. Among Phase 1b patients, 13 patients (68.4%) had received surgery as the only prior treatment for their disease, while the other 6 patients did not have any prior treatment. Phase 1b patients had an ECOG performance status of 0 or 1 (n=15 [78.9%]), and a pathological diagnosis of glioblastoma (n=17 [89.5%] patients) at study entry.

The safety and tolerability of a Phase 2a dose of galunisertib were evaluated during Phase 1b study by giving sequentially two dose levels of galunisertib (dose escalation from 160 mg/day [n=10] to 300 mg/day [n=9]) to patients, in combination with radiochemotherapy (Supplemental Fig. 1a). The dose of galunisertib at 300 mg/day did not increase the toxicity profile compared to the dose of 160 mg/day, and no overlapping toxicity was observed by the combination of galunisertib with radiochemotherapy (Supplemental Table 3). The main reasons for study treatment discontinuation were progressive disease (13/19 patients [68.4%]), AEs (2 [10.5] with one case of rash maculo-papular, and one pulmonary embolism), sponsor decision (1 [5.2%]), and physician decision (2 [10.5%]) (Supplemental Fig. 1). While the majority of doses of galunisertib were provided as planned, 3 patients had a dose reduction of galunisertib during the first two cycles of Phase 1b treatment due to AEs (decreased of platelet count, dermatitis acneiform and monoparesis; data not shown). The dose adjustment in TMZ was mostly due to decreased platelet count (data not shown).

Among the 19 patients from Phase 1b, 10 SAEs occurred in 6 patients. There were no pathological laboratory results consistent with myocardial infarct (i.e., negative troponin I), significant cardiac insufficiency (i.e, increased BNP levels over 3 consecutive blood draws at 3 times the baseline value and above the upper limit of normal [ULN]), or other cardiac markers of aneurysm formation (i.e., cystatin C), or vascular inflammation (i.e, hs-CRP). There was one case in which central echocardiographic assessment detected a "moderate" mitral valve regurgitation, which did not deteriorate during the course of treatment. The most frequent drug-related treatment-emergent adverse events (TEAEs) experienced by Phase 1b patients were nausea (36.8%), thrombocytopenia (36.8%), fatigue (31.6%), alopecia (26.3), lymphocyte count decrease (21.1%) (Supplemental Table 2). The drug-related TEAEs occurring at CTCAE ≥ grades 3 were platelet count decreased (31.6%, grade 3; 5.3%, grade 4), lymphocyte count decreased (10.5%, grade 3; 5.3%, grade 4), white blood cell count decreased (10.5% grade 3;

5.3%, grade 4), rash maculo-papular (10.5%, grade 3), neutrophil count decreased (5.3%, grade 3; 5.3%, grade 4), tumor haemorrhage (5.3%, grade 3), anemia (5.3%, grade 3), alanine aminotransferase increased (5.3%, grade 3). One patient experienced pancytopenia and, after bone marrow biopsy, was diagnosed with myeloablative marrow aplasia, showing markedly hypocellular (less than 5%) bone marrow trilineage aplasia, including grade 4 thrombocytopenia. Thrombocytopenia was attributed mainly to the TMZ administration, while not excluding some possible (not likely) association with LY2157299. There were no dose-limiting toxicities (DLT) and no clinically meaningful cardiotoxicities observed. None of the patients met the definition of DLT, per protocol and investigator/sponsor decision. Given that TMZ in combination with radiation carries a risk of approximately 19% grade 3/4 thrombocytopenia, and 13% grade 2 rash, the observed event rate in Phase 1b was not unexpectedly high [3,4].

The median OS for all 19 Phase 1b patients was 17.5 months (95% CI: 10.1, 22.7) (data not shown).

The PK data of Phase 1b were consistent with the previously described PK analysis of galunisertib suggesting that the PK profile of galunisertib was not altered when combined with TMZ and radiation.

To conclude, based on overall toxicity and PK information from Phase 1b, the dose of 300 mg/day of galunisertib was selected for the Phase 2a part of the study.

References

1. Capper D, von Deimling A, Brandes AA, et al (2017) Biomarker and Histopathology Evaluation of Patients with Recurrent Glioblastoma Treated with Galunisertib, Lomustine, or the Combination of Galunisertib and Lomustine. Int J Mol Sci 18(5):995.

2. Wen PY, Macdonald DR, Reardon DA, et al (2010) Updated response assessment criteria for high-grade gliomas: response assessment in neuro-oncology working group. J Clin Oncol 28(11):1963-1972.

3. Gerber DE, Grossman SA, Zeltzman M, Parisi MA, Kleinberg L (2007) The impact of thrombocytopenia from temozolomide and radiation in newly diagnosed adults with high-grade gliomas. Neuro Oncol 9(1):47-52.

4. Addeo R, Caraglia M, Faiola V, et al (2007) Concomitant treatment of brain metastasis with whole brain radiotherapy [WBRT] and temozolomide [TMZ] is active and improves quality of life. BMC Cancer 7:18.

Supplemental Table 1. Phase 2a: Summary of treatment response

Response, n (%), [90% CI]	Gal + TMZ/RTX N=40	TMZ/RTX N=16
Complete response (CR)	3 (7.5) [2.1, 18.3]	0 (0.0)
Partial response (PR)	0 (0.0)	0 (0.0)
Stable disease (SD)	29 (72.5) [58.6, 83.7]	9 (56.3) [33.3, 77.3]
Progressive disease (PD)	7 (17.5) [8.5, 30.4]	3 (18.8) [5.3, 41.7]
Unknown	1 (2.5)	4 (25.0)
Response rate (CR+PR)	3 (7.5) [2.1, 18.3]	0 (0.0)
Disease control rate (CR+PR+SD)	32 (80.0) [66.8, 89.6]	9 (56.3) [33.3, 77.3]

Abbreviations: Gal = galunisertib; TMZ = temozolomide; RTX = radiation

Supplemental Table 2. Phase 1b patient demographics and baseline characteristics

Phase 1b patient Characteristics	Gal (160) + TMZ/RTX N = 10	Gal (300) + TMZ/RTX N = 9	Total N = 19
**Gender**, Male	7 (70)	5 (55.6)	12 (63.2)
**Age,** years, mean, (SD)	52.3 (8.1)	57.6 (12.3)	54.8 (10.4)
**Race**, White	10 (100)	9 (100)	19 (100)
**ECOG** PS			
0	7 (70)	1 (11.1)	8 (42.1)
1	0 (0.0)	7 (77.8)	7 (36.8)
Missing	3 (30)	1 (11.1)	4 (21.1)
**Basis of initial pathological diagnosis**			
Histopathological	9 (90)	9 (100)	18 (94.7)
Mixed: cytological/histopathological	1 (10)	0 (0)	1 (5.3)
**Study entry pathological diagnosis**			
Glioblastoma	8 (80)	9 (100)	17 (89.5)
Glioma, astrocytoma	1 (10)	0 (0.0)	1 (5.3)
Glioma, oligodendroglioma	0 (0.0)	0 (0.0)	0 (0.0)
Mixed	1 (10)	0 (0.0)	1 (5.3)
Glioblastoma	1 (10)	0 (0.0)	1 (5.3)
Glioma, astrocytoma	1 (10)	0 (0.0)	1 (5.3)
**Prior treatment**			
Surgery	8 (80)	5 (55.6)	13 (68.4)

To note: data given as No (%) unless otherwise indicated
Abbreviations: Gal = galunisertib; TMZ = temozolomide; RTX = radiation; SD = standard deviation; ECOG PS = Eastern Cooperative Oncology Group performance status

Supplemental Table 3. Drug-related TEAEs occurring in ≥10% of Phase 1b patients in either arm by system organ class and preferred term (patient on therapy)

	Phase 1b		
	TMZ/RTX		
	Gal. 160 mg N=10	Gal. 300 mg N = 9	Total N =19
**Patients with ≥ 1 drug related TEAE**	10 (100)	8 (88.9)	18 (94.7)
**Gastrointestinal Disorders**			
Nausea	6 (60.0)	1 (11.1)	7 (36.8)
Vomiting	0 (0.0)	3 (33.0)	3 (15.8)
Constipation	2 (20.0)	0 (0.0)	2 (10.5)
Dyspepsia	0 (0.0)	1 (11.1)	1 (5.3)
**General disorders and administration site conditions**			
Fatigue	3 (30.0)	3 (33.3)	6 (31.6)
Asthenia	2 (20.0)	0 (0.0)	2 (10.5)
**Skin and subcutaneous tissue disorders**			
Alopecia	4 (40.0)	1 (11.1)	5 (26.3)
Rash maculo-papular	2 (20.0)	1 (11.1)	3 (15.8)
Dermatitis acneiform	1 (10.0)	2 (22.2)	3 (15.8)
**Investigations**			
Lymphocyte count decreased	2 (20.0)	2 (22.2)	4 (21.1)
Weight decreased	1 (10.0)	2 (22.2)	3 (15.8)
ALT	1 (10.0)	1 (11.1)	2 (10.5)
AST	1 (10.0	1 (11.1)	2 (10.5)
Lipase increased	1 (10.0)	1 (11.1)	2 (10.5)
**Blood and Lymphatic system disorders**			
Thrombocytopenia	5 (50.0)	2 (22.2)	7 (36.8)
Lymphopenia	1 (10.0)	2 (22.2)	3 (15.8)
Anaemia	2 (20.0)	0 (0.0)	2 (10.5)
Neutropenia	1 (10.0)	1 (11.1)	2 (10.5)
Leukopenia	0 (0.0)	2 (22.2)	2 (10.5)
**Metabolism and nutrition disorders**			
Decreased appetite	2 (20.0)	0 (0.0)	2 (10.5)
**Injury, poisoning and procedural complications**			
Radiation skin injury	3 (30.0)	0 (0.0)	3 (15.8)

To note: data given as n (%) unless otherwise indicated
Abbreviations: Gal = galunisertib; TMZ = temozolomide; RTX = radiation; ALT = alanine aminotransferase; AST = aspartate aminotransferase; TEAE = treatment-emergent adverse events

Supplemental Table 4. Phase 2a: summary of histopathological evaluation

	Result n (%)	TMZ+RTX N=16[a]	Gal + TMZ+RTX N=40[a]	All Patients N=56[a]	Excluding 2 IDH1 R132H positive patients N=54
**Initial Diagnosis**					
	Glioblastoma	14 (100%)	34 (94%)	48 (96%)	47 (98%)
	Glioma, astrocytoma	0	2 (5.6%)	2 (4%)	1 (2%)
	Missing	2	4	6	6
**IDH1 R132H**					
	Positive	0	2 (6%)	2 (4%)	-
	Negative	13 (93%)	33 (94%)	46 (94%)	-
	Not evaluable	1 (7%)	0	1 (2%)	-
	Missing	2	5	7	-
**Assessment of differentiation**	Perinuclear halos	1 (6%)	2 (5%)	3 (5%)	3 (6%)
	Fibrillary astrocytoma-like foci	0	0	0	0
	Small cell astrocytoma-like foci	2 (13%)	6 (16%)	8 (15%)	8 (16%)
	Polar spongioblastic foci	0	0	0	0
	Protoplasmic astrocytoma-like foci	0	0	0	0
	Minigemistocytes	0	0	0	0
	Classic gemistocytes	0	3 (8%)	3 (6%)	3 (6%)
	Giant cells	2 (13%)	3 (8%)	5 (9%)	5 (10%)
	PNET-like	2 (13%)	1 (3%)	3 (6%)	3 (6%)
	Sarcoma-like	0	1 (3%)	1 (2%)	1 (2%)
	Microcysts	0	0	0	0
	Mucoid degeneration	0	1 (3%)	1 (2%)	1 (2%)
	Calcifications	0	0	0	0
	Missing	0	2[b]	2	2
**Cellular density**					
	High (like PNET)	3 (19%)	1 (3%)	4 (7%)	4 (8%)
	Medium (like classical glioblastoma)	13 (81%)	36 (95%)	49 (91%)	47 (90%)
	No	0	1 (3%)	1 (2%)	1 (2%)
	Missing	0	2	2	2

	Result n (%)	TMZ+RTX N=16[a]	Gal + TMZ+RTX N=40[a]	All Patients N=56[a]	Excluding 2 IDH1 R132H positive patients N=54
**Vessel structure[c]**					
	Abnormal number of vessels	15 (100%)	34 (89%)	49 (92%)	48 (94%)
	Any endothelial hypertrophy	15 (100%)	36 (100%)	51 (100%)	50 (100%)
	Glomeruloid blood vessel	8 (53%)	21 (58%)	29 (57%)	28 (56%)
	Multi-layering blood vessel	14 (93%)	35 (97%)	49 (96%)	48 (96%)
	Vascular abnormalities	15 (100%)	35 (95%)	50 (96%)	49 (98%)
	Vessel thrombosis	11 (73%)	18 (47%)	29 (55%)	29 (57%)
**Tumor necrosis**					
	Yes, with pseudopalisading	6 (40%)	9 (24%)	15 (28%)	15 (29%)
	Yes, without pseudopalisading	7 (47%)	20 (53%)	27 (51%)	26 (51%)
	No	2 (13%)	9 (24%)	11 (21%)	10 (20%)
	Missing	1	2	3	3
**Mitotic scoring**					
	≤5 Mitoses (per 10 Highpower fields)	4 (27%)	11 (29%)	15 (28%)	14 (27%)
	6-20 Mitoses (per 10 Highpower fields)	7 (47%)	21 (55%)	28 (53%)	27 (53%)
	>20 Mitoses (per 10 Highpower fields)	4 (27%)	6 (16%)	10 (19%)	10 (20%)
	Missing	1	2	3	3
**Nuclear abnormalities**					
	Medium (like classical glioblastoma)	0	1 (3%)	1 (2%)	1 (2%)
	Medium (abnormal nuclear shape)	13 (81%)	31 (82%)	44 (81%)	42 (81%)
	High (bizarre nuclei)	3 (19%)	6 (16%)	9 (17%)	9 (17%)
	Missing	0	2	2	2
**CD3 parenchymal lymphocytic infiltrate**					
	≤1%	7 (54%)	15 (45%)	22 (48%)	20 (45%)
	2-4%	6 (46%)	15 (45%)	21 (46%)	21 (48%)
	≥5%	0	2 (6%)	2 (4%)	2 (5%)
	Positive	0	1 (3%)	1 (2%)	1 (2%)
	Missing	3	7	10	10
**CD3 perivascular lymphocytic infiltrate**					
	None[d]	5 (38%)	7 (21%)	12 (26%)	11 (25%)
	Slight[e]	5 (38%)	15 (45%)	20 (43%)	19 (43%)
	Prominent[f]	3 (23%)	10 (30%)	13 (28%)	13 (30%)
	Positive	0	1 (3%)	1 (2%)	1 (2%)
	Missing	3	7	10	10

	Result n (%)	TMZ+RTX N=16[a]	Gal + TMZ+RTX N=40[a]	All Patients N=56[a]	Excluding 2 IDH1 R132H positive patients N=54
**Ki67**					
	≤5%	0	1 (3%)	1 (2%)	1 (2%)
	6-10%	3 (23%)	11 (34%)	14 (31%)	14 (33%)
	11-20%	7 (54%)	13 (41%)	20 (44%)	20 (47%)
	>20%	3 (23%)	7 (22%)	10 (22%)	8 (19%)
	Missing	3	8	11	11
**Glial fibrillary acid protein**	Cyto Total Detected (H score[g] >0)	13 (100%)	32 (100%)	45 (100%)	43 (100%)
	Cyto H score[g] Median (25th perc, 75th perc)	60 (50, 110)	155 (103, 195)	140 (70, 180)	140 (70, 180)
	Missing	3	8	11	11
**pSMAD2**					
	Cyto Total Detected (H score[g] >0)	4 (31%)	10 (29%)	14 (29%)	14 (30%)
	Cyto H score[g] Median (25th perc, 75th perc)	0 (0, 20)	0 (0, 20)	0 (0, 20)	0 (0, 20)
	Nuclei Total Detected (H score[g] >0)	13 (100%)	35 (100%)	48 (100%)	46 (100%)
	Nuclei H score[g] Median (25th perc, 75th perc)	100 (80, 175)	120 (55, 165)	120 (65, 173)	125 (70, 175)
	Missing	3	5	8	8

[a]Denominator for the percentage calculation is the number of randomized patients with tumor sample for which an evaluable result was obtained (not number of patients dosed). Percentages are rounded to nearest integer

[b]Missing patients for Small cell astrocytoma-like foci is 3

[c]Differing numbers of missing data for Vessel Structure: 1 patient missing for all variables in TMZ+RTX arm and ranging from 2-4 for the Gal + TMZ+RTX arm

[d]≤4 perivascular positive cells per vessel

[e]≥1 vessel with ≥5 and <30 positive

[f]≥1 vessel with ≥5 and ≥30 positive perivascular cells

[g]Total possible H-score=300

Abbreviations: Cyto = cytoplasm; perc = percentile

Supplemental Fig. 1. Phase 1b: Patient dispositions from treatment.

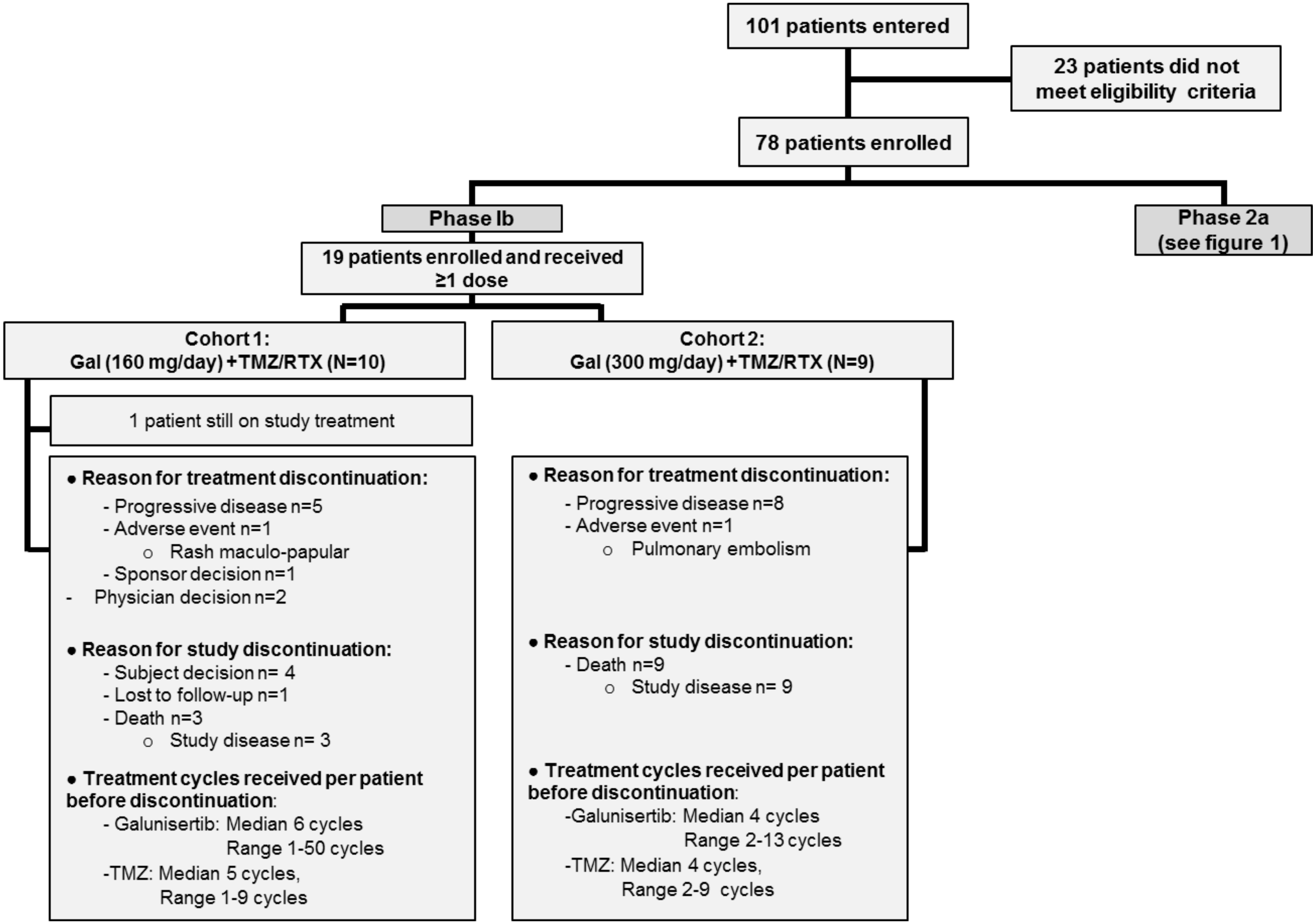

Supplemental Fig. 2. Study (A) and treatment (B) design of galunisertib plus chemoradiotherapy in Phase 1b/2a patients with newly diagnosed glioblastoma.

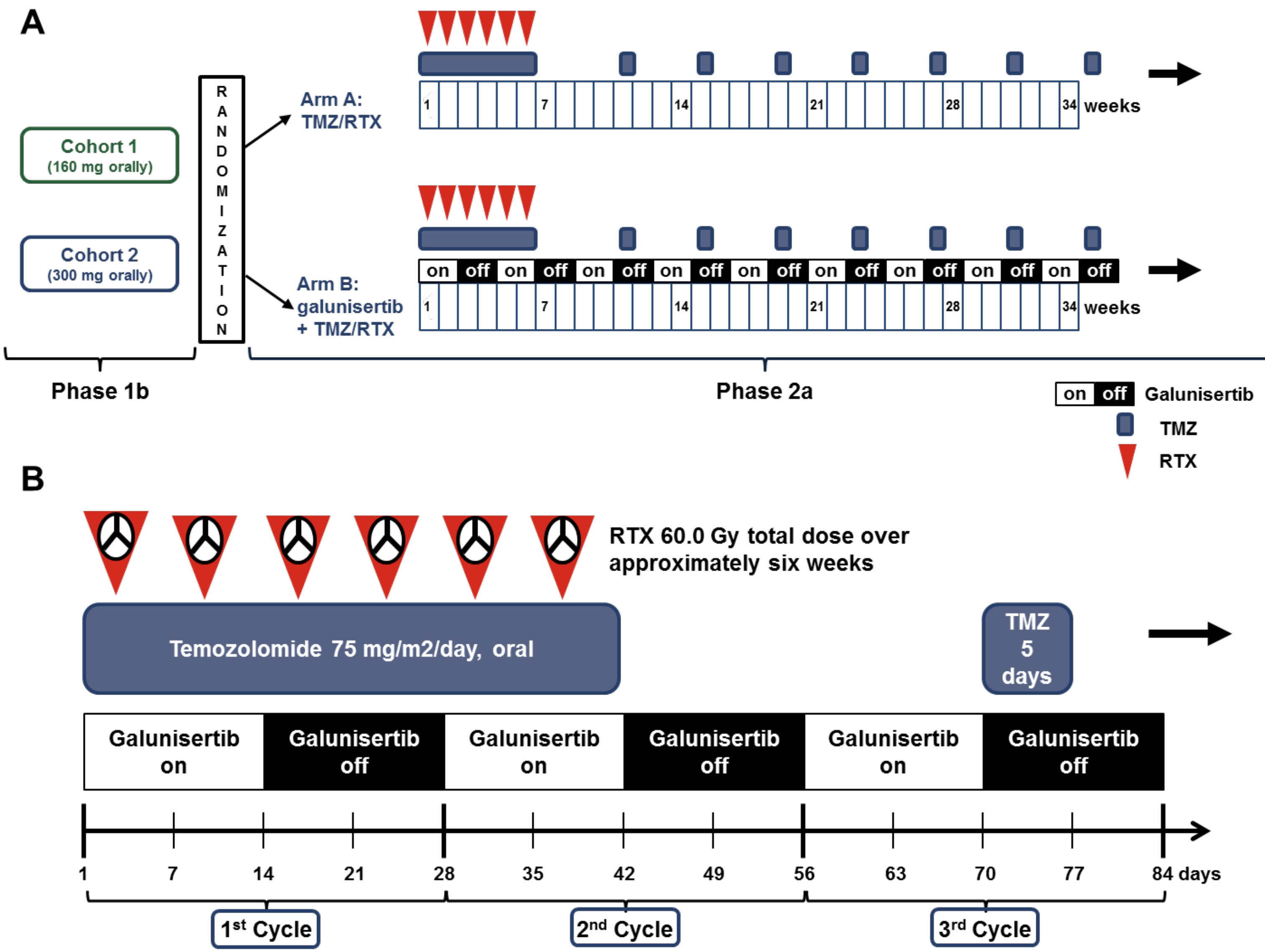

Supplemental Fig. 3. Correlation of OS or PFS and baseline flow cytometry markers in Phase 2a. Hazard ratios (HR) for the correlation of OS (A) or PFS (B) with baseline flow cytometry markers where HR of 1 means no effect. YKL-40 expression was not assessed by flow cytometry.

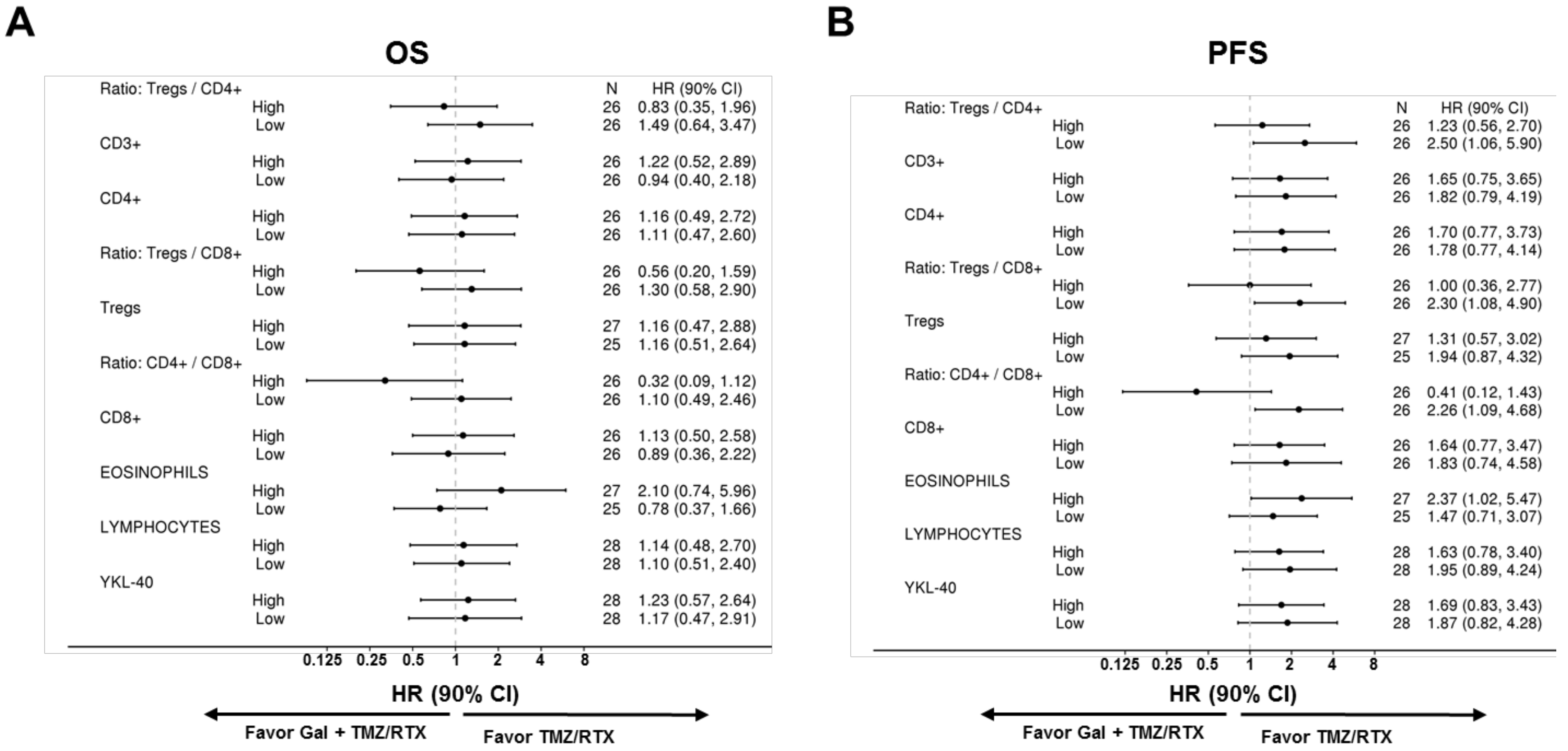

Supplemental Fig. 4. Summary of galunisertib pharmacokinetic parameters following multiple oral doses of galunisertib. (A) Galunisertib noncompartmental pharmacokinetic parameters following multiple oral doses in Phase 1b at steady state day 14, assessed over 48 hours (Cycle 1) and 24 hours (Cycles 2 and 3) after last dose. Abbreviations: t_{max} is the time of maximum observed drug concentration. C_{max} is the maximum observed drug concentration; AUC(0-∞) is the area under the concentration versus time curve from time 0 extrapolated to infinity; h=hours. (B) Dose normalized plasma galunisertib AUC 0-∞,ss in Cycle 1, 2 and 3 following twice daily doses of either 80 or 150 mg galunisertib. Abbreviation: AUC_{inf} is the area under the plasma concentration-time curve extrapolated to infinity. (C) Phase 1b and Phase 2a PK data for all days and cycles as defined in the PK sampling schedule were combined in a dataset, the observations were dose normalized and plotted by cycle. Plasma galunisertib concentration by Cycle of treatment at 160 mg and 300 mg per day (all PK data Phase 1b and Phase 2a). Cycle 1 (n=57 [160 mg, n=10; 300 mg, n=47]; n=539 observations Phase 1a and 2b); Cycle 2 (n=51 [160 mg, n=9; 300 mg, n=42]; n=369 observations Phase 1a and 2b); Cycle 3 (n=17 [160 mg, n=9; 300 mg, n=8]; n=151 observations Phase 1a only).

A

	Galunisertib noncompartmental pharmacokinetic					
	160 mg Galunisertib			300 mg Galunisertib		
	Cycle 1	Cycle 2	Cycle 3	Cycle 1	Cycle 2	Cycle 3
N	10	8	5	8	8	7
C_{max} (ng/ml)	653 (91)	580 (75)	728 (67)	1620 (70)	1160 (48)	1550 (47)
t_{max} (h) Range	1.29 (0.5-3.0)	1.13 (0.5-3.0)	0.75 (0.48-2.0)	1.21 (0.5-3.0)	1.5 (0.5-2)	1 (0.5-2.08)
AUC(0-∞) (ng•h/ml)	1860 (93)	1910 (89)	1920 (104)	6290 (70)	4940 (35)	5510 (27)

B

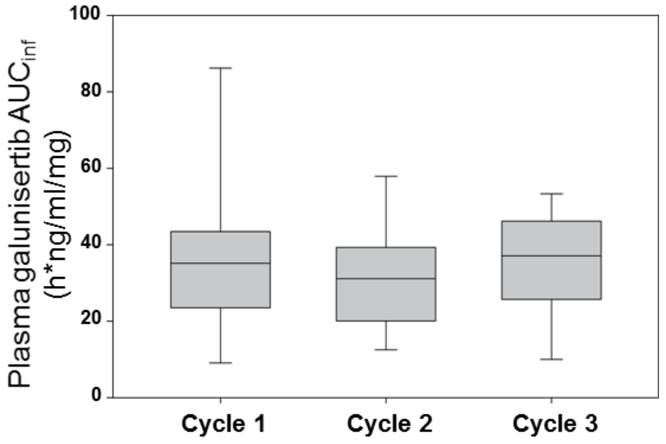

C

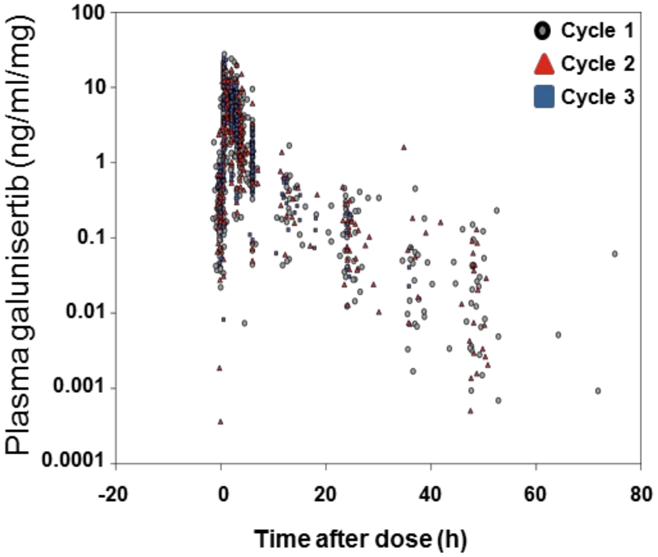

Supplement: Supplementary file 1 — (PDF 661 kb) [file 10637_2020_910_MOESM1_ESM.pdf]
